# Biomarkers for Frailty and Functional Decline in Aging

**DOI:** 10.1093/geroni/igaf122.230

**Published:** 2025-12-31

## Abstract

In this session research will be presented on biological and clinical biomarkers for frailty and functional decline in older adults.

